# Insectivorous bats are less active near freeways

**DOI:** 10.1371/journal.pone.0247400

**Published:** 2021-03-10

**Authors:** Manisha Bhardwaj, Kylie Soanes, José J. Lahoz-Monfort, Linda F. Lumsden, Rodney van der Ree

**Affiliations:** 1 School of BioSciences, University of Melbourne, Melbourne, Victoria, Australia; 2 School of Ecosystem and Forest Sciences, University of Melbourne, Melbourne, Victoria, Australia; 3 Department of Environment, Land, Water and Planning, Arthur Rylah Institute for Environmental Research, Heidelberg, Victoria, Australia; Michigan State University, UNITED STATES

## Abstract

Traffic disturbances (i.e. pollution, light, noise, and vibrations) often extend into the area surrounding a road creating a ‘road-effect zone’. Habitat within the road-effect zone is degraded or, in severe cases, completely unsuitable for wildlife, resulting in indirect habitat loss. This can have a disproportionate impact on wildlife in highly modified landscapes, where remaining habitat is scarce or occurs predominantly along roadside reserves. In this study, we investigated the road-effect zone for insectivorous bats in highly cleared agricultural landscapes by quantifying the change in call activity with proximity to three major freeways. The activity of seven out of 10 species of bat significantly decreased with proximity to the freeway. We defined the road-effect zone to be the proximity at which call activity declined by at least 20% relative to the maximum detected activity. The overall road-effect zone for bats in this region was 307 m, varying between 123 and 890 m for individual species. Given that this road-effect zone exceeds the typical width of the roadside verges (<50 m), it is possible that much of the vegetation adjacent to freeways in this and similar landscapes provides low-quality habitat for bats. Without accounting for the road-effect zone, the amount of habitat lost or degraded due to roads is underestimated, potentially resulting in the loss of wildlife, ecosystem services and key ecosystem processes (e.g. predator-prey or plant-pollinator interactions) from the landscape. We suggest all future environmental impact assessments include quantifying the road-effect zone for sensitive wildlife, in order to best plan and mitigate the impact of roads on the environment. Mitigating the effects of new and existing roads on wildlife is essential to ensure enough high-quality habitat persists to maintain wildlife populations.

## Introduction

Roads and traffic are prominent features of most landscapes [1] and can have numerous negative impacts on wildlife, such as road mortality, barrier effects, and habitat loss and degradation [2–5]. Strategies to reduce the impacts of roads on wildlife often focus on identifying and mitigating road mortality and barrier effects [6]. However, the ‘road-effect zone’ may be just as detrimental to the ability of a species to persist in a landscape as other effects [3, 7–9], and requires consideration while planning and designing road projects [6].

The road-effect zone is the distance to which the ecological impacts of roads extend into the surrounding habitat [3, 7]. Road-effect zones have been quantified for birds [10, 11], mammals [e.g. 12–14], amphibians [e.g. 15–17] and reptiles [e.g. 18, 19]. The road-effect zone typically reflects a negative impact on wildlife [20], as within this zone, habitat is degraded and indirectly lost, evident through decreased activity or survivorship of individuals, and smaller populations [e.g. 3, 17, 20] compared to further away from the road. The size of the road-effect zone can be large: up to 1 km for some birds (e.g. Passeriformes and Piciformes) and up to 5 km for some mammals (e.g. Rodentia and Artidactyla) [20]. Habitat degradation in the road-effect zone can be due to a number of factors, such as noise and light pollution, unsuitable vegetation type, and chemical pollution [3, 21]. Without accounting for the road-effect zone, the amount of habitat lost or degraded due to roads will be underestimated, which can potentially result in the loss of wildlife, ecosystem services and key ecosystem processes (e.g. predator-prey or plant-pollinator interactions) from the landscape [22]. Understanding the size and the severity of the road-effect zone on a wide range of species will improve decision-making regarding strategies to avoid, minimize, mitigate and compensate the impact of roads on wildlife.

Many species of bats (order: Chiroptera) avoid the habitat adjacent to major roads and freeways [14, 23, 24]. This may be due to a lack of canopy cover [25, 26] or due to disturbances from the road and traffic, such as light and noise [e.g. 24, 27–29]. The size of the road-effect zone for bats can be highly variable—i.e. up to 500 m from a freeway for some species [23, 30], or up to 5,000 m for others [14, 31]. In highly cleared landscapes, such as agricultural areas, a large proportion of the habitat exists in verges adjacent to high-traffic freeways and roads. This means that the road-effect zone can potentially cover the entire extent of available habitat and drive bats out of the landscapes. Given their high trophic levels, widespread mobility and their responsiveness to anthropogenic stressors, bats are considered bioindicator species and in agricultural landscapes they also offer key ecosystem services such as pest-control and pollination [32–34]. Thus the loss of bats from such landscapes can be detrimental to ecosystem health, however the severity of the road-effect zone on bats in highly cleared agricultural landscapes is understudied and unclear [e.g. 30]. As a result, decision-makers may face uncertainty in how to best manage this landscape to reduce the negative impacts of roads on bats, and preserve bat presence.

The aim of this study is to quantify the road-effect zone for insectivorous bats (henceforth referred to as ‘bats’) in the agricultural landscapes of south-eastern Australia. Bats in this region can persist in highly cleared landscapes as long as there is some mature vegetation for them to roost in and forage around [35]. However, a negative road-effect zone may hinder their ability to use mature trees in freeway verges. Using these results, we aim to suggest potential causes and mitigation options for the road-effect zone for bats in highly cleared agricultural landscapes.

## Materials and methods

Research was conducted under Scientific Permit 10006093 granted by the Department of Environment, Land, Water & Planning.

### Study area

**Fig 1:**
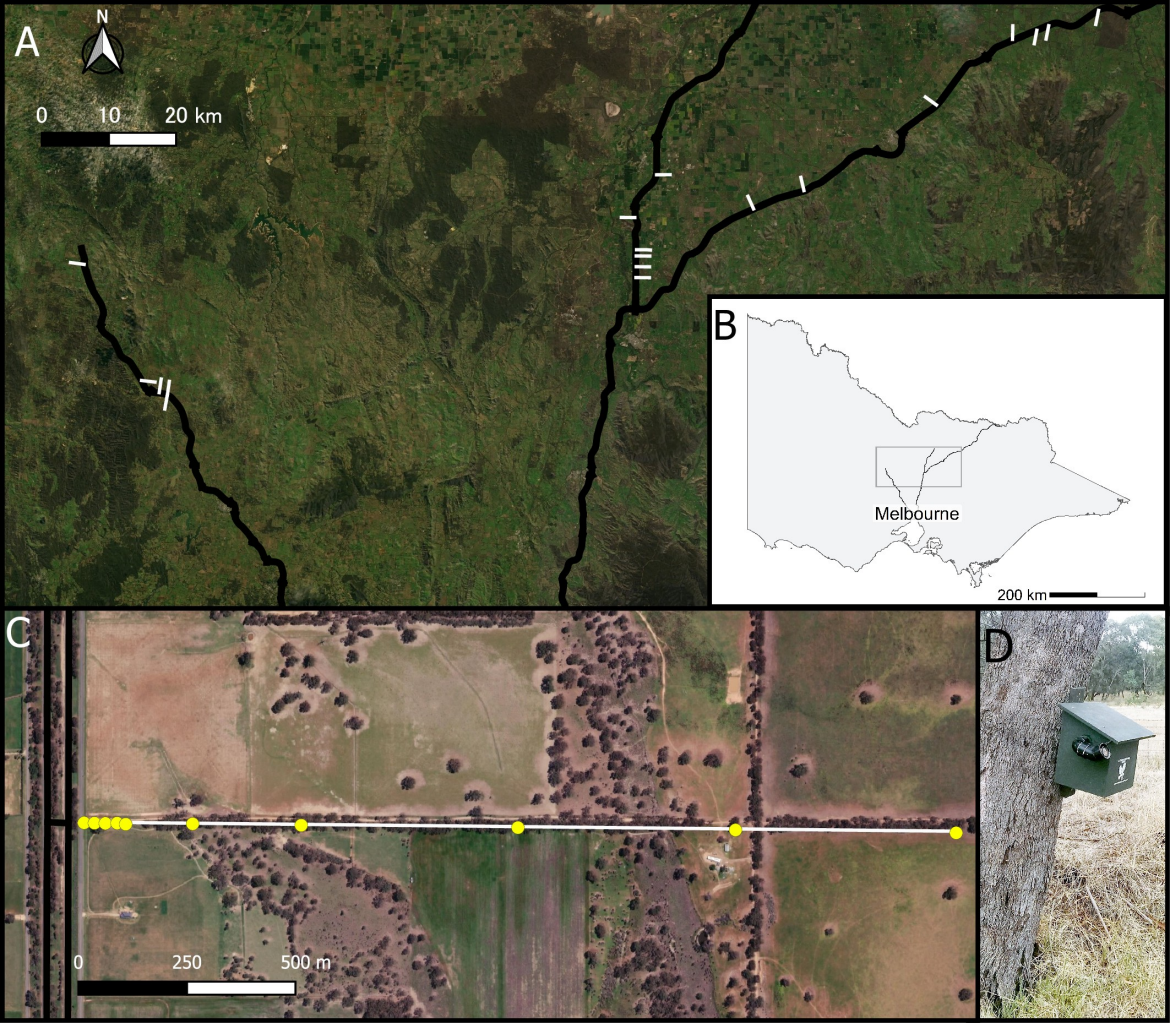
Sites and detector installations. (A) Map of the study sites showing the three focal freeways in black, study transects in white. (B) Map of Victoria, Australia showing the three focal freeways in black. The grey box corresponds to the extent of A. (C) Example of a transect from an aerial view, showing the dual carriage freeway in black on the left of the frame and the transect in white. The yellow circles along the transect show detector placements at 10 m, 25 m, 50 m, 75 m, 100 m, 250 m, 500 m, 1000 m, 1,500 m and 2,000 m away from the freeway. (D) Side view of a wooden nest box with an Anabat detector within it, affixed to a tree approximately 1 m from the ground. The microphone was placed within a PVC pipe, oriented at 45° towards the sky. A and C were produced in QGIS (3.16.1-Hannover) using the base map layer, ESRI Satellite (ArcGIS/World_Imagery; Sources: Esri, DigitalGlobe, GeoEye, i-cubed, USDA FSA, USGS, AEX, Getmapping, Aerogrid, IGN, IGP, swisstopo, and the GIS User Community).

This study was conducted in the predominantly agricultural landscape of central Victoria, Australia. This region is heavily cleared with remnants of heathy dry forest, grassy woodlands and box-ironbark forest [36], mostly existing in small patches, or linear strips such as roadside and freeway verges (Fig 1). The agricultural land is predominately used for grazing, with some opportunistic crop production [37]. There are three major freeways in this region: Hume Freeway, Goulburn Valley Freeway and Calder Freeway. Within our study area, the freeways have two lanes of traffic in each direction and each carriageway is 12 m wide, separated by a vegetated median approximately 5–20 m in width. The maximum speed limit is 110 km/h. The annual average daily traffic volume (in one direction) averages 6,140 (range 5,800–6,300) vehicles/day along the Hume Freeway, 4,460 (range 3,700–4,800) vehicles/day along the Goulburn Valley Freeway and 6,720 (range 5,500–9,100) vehicles/day along the Calder Freeway [38]. Vegetated verges along the edges of the freeways are on average 28 m in width (range 0–50 m) and typically consist of eucalypt woodland.

Each freeway is intersected by numerous linear strips of woodland vegetation which occur along, for example, property boundaries, waterways and farming roads (Fig 1). The vegetation in these linear strips can provide suitable habitat [35, 39–42] and commuting routes [42–45] for bats in this region. Small farming roads (i.e. single-lane, fewer than 100 vehicles per day) intersected the freeway perpendicularly and extended at least 2,000 m from the freeway, providing ideal “transects” for studying road-effect zone for bats. We surveyed bats along 18 transects distributed among the three freeways (Calder Freeway: n = 5, between -36.92150° S, 144.22181° E, to -37.09743° S, 144.35961° E; Goulburn Valley Freeway: n = 6, between -36.80396° S, 145.17779° E, and -36.94174° S, 145.14383° E; Hume Freeway: n = 7, between -36.57926° S, 145.91285° E and -36.84920° S, 145.34005° E; Fig 1). Transects along the same highway were between 1 and 19 km from the nearest transect (12 transects were within 5 km of the nearest neighbouring transect).

### Recording and analysing bat call activity

We collected data in the austral summer between December 2014 and February 2015, when this region is predominately hot and dry with low to moderate humidity. Given the variability of the road-effect zone previously documented for bats, we chose to collect data in short intervals up to 500 m from the freeway as well as at larger intervals up to 2,000 m from the freeway (i.e. the length of the transects), to improve our chances of detecting a road-effect zone for bats. Data was collected at 10 distance intervals from the freeway along each transect: 0–10 m, 25 m, 50 m, 75 m, 100 m, 250 m, 500 m, 1000 m, 1,500 m and 2,000 m (Fig 1). The distance of the placement of the detector within the first interval (0–10 m) depended on where there was a suitable tree to install the detector on.

At each distance interval, we recorded bat calls using Anabat SD1 and SD2 model detectors, with unidirectional microphones that had been calibrated to have similar sensitivities (Titley Electronics, Ballina, New South Wales, Australia). To reduce the risk of theft, interference and damage, we disguised the detectors by placing them in wooden nest boxes. On the sides of the nest boxes, we attached a PVC pipe, oriented it 45° towards the sky and secured the microphone within the pipe opening (Fig 1). All nest boxes were affixed to trees (approximately 1 m from the ground), with the microphones pointing away from the freeway. We recorded calls at each transect for two consecutive nights, commencing half an hour before sunset and ending half an hour after sunrise, with all points along a transect surveyed concurrently. We surveyed two transects each night. Due to equipment malfunctions and absence of trees suitable for attaching the nest box, we were unable to collect a sample at each distance every night. In total, we collected 280 samples (out of a potential total of 360), with 21–36 samples at each distance out of a potential maximum of 36 (0–10 m: n = 29; 25m: n = 21; 50 m: n = 22; 75 m: n = 32; 100 m, n = 30; 250 m: n = 26; 500 m: n = 36; 1000 m: n = 28; 1,500 m: n = 32; 2,000 m: n = 24).

We identified the recorded Anabat zero crossing call sequences (henceforth referred to as “calls”) to species using the automated AnaScheme Bat Call Analysis System (Version 1.0) [46, 47]. A key was previously developed for this region using locally collected reference calls, which, when tested on an independent set of reference calls, was found to accurately identify 72% of the reference calls, with no mis-identifications while the remaining 28% were identified as ‘unknowns’ [35]. We used this key to identify the calls we collected in this study. To further reduce misidentifications, we only attempted species identification when there were five or more valid pulses in the call and we deemed calls successfully identified when >50% of the pulses were assigned to the same species [35]. We grouped calls from *Nyctophilus geoffroyi*, *Nyctophilus gouldi* and *Myotis macropus*, into a ‘*Nyctophilus-Myotis* complex’ because their calls are difficult to differentiate reliably from one another. We also visually confirmed any files identified as *Austronomus australis* as the key was prone to misattributing insect or background noise to this species. It was not possible to determine the number of individuals from the call data so our analysis and results reflect species activity rather than population sizes.

### Environmental variables that may influence bat activity

Bat activity along transects could have been influenced by the amount and type of habitat available along the transect as well as the landscape surrounding the transect [30, 35, 48]. To minimize these effects, all transects were tree-lined on both sides of the road (transect verges were, on average, 8 m wide and ranged 1–30 m wide) and adjacent to agricultural fields and scattered residences.

The size of the trees and structure of the vegetation around each detector may influence the activity and presence of bats regardless of the distance from the freeway. Bats may prefer large trees (diameter greater than 30 cm at breast height) because they tend to have more hollows than smaller trees, which provides greater roosting opportunities [41], and have more flowers, greater peeling bark and greater canopy cover, which all support more insect prey [35]. Therefore, we recorded the species and size (diameter at breast height) of all trees and visually assessed percentage canopy cover (to the nearest 10%) within a 10 m radius of each detector. A 10 m radius was adopted to avoid overlapping sampling areas because the first 5 detectors were 25 m from the adjacent detector.

Finally, to account for any influence that temperature and nightly variation in weather conditions can have on the presence and activity of bats [49–52], we obtained daily minimum temperature data (typically recorded overnight) from the Australian Government, Bureau of Meteorology (http://www.bom.gov.au/climate/data; weather stations were within 20 km of the sites).

### Statistical analysis

To explore the change in activity of bats with distance from the freeway, we fitted Poisson regression models, using the number of calls per survey night (i.e. call rate, *R*_*i*_) as the response variable. So, for each data point, i (each distance along the transect, per night):

$$R_{i}∼Poisson\left(\lambda_{i}\right)$$

$$\log\left(\lambda_{i}\right)=\beta_{0}+\beta_{1}D_{i}+\beta_{2}T_{i}+\beta_{3}L_{i}+\beta_{4}C_{i}+\varepsilon_{x\left(i\right)}$$

where λ_i_ was the mean call rate. *D*_*i*_ was the distance from the freeway at which the detector was placed along the transect. More specifically, we used the log function *D*_*i*_ = log(*distance* + 1) to reflect the diminishing strength with distance, as expected from a potential road-effect, and added 1 to the distance to avoid negative log numbers for distance zero. We compared this model using a linear relationship with distance (standardised) and found that the log approach provided better fitting models (either lower or similar DIC values).

To account for nightly variation in environmental conditions we included daily minimum temperature (*T*_*i*_), and the number of large trees (*L*_*i*_) and canopy cover (*C*_*i*_) within a 10 m radius of the detector. Finally, we included a random effect term for the transect, *ε*_*x*(*i*)_. Activity did not differ significantly among the freeways, so we combined the data from the three freeways. Daily minimum temperature and canopy cover were standardized around the mean. The intercept, *β*_0_, represents the baseline (0 m from the freeway, mean temperature, no large trees, mean canopy cover). All model fitting was conducted within a Bayesian framework of inference using Markov Chain Monte Carlo (MCMC) sampling, by calling JAGS 4.1.0 [53] from R [54] using package R2jags [55]. We ran three MCMC chains for each parameter, keeping 300,000 iterations after discarding a burn-in of 100,000, with a thinning of 10 to reduce the size of resulting files. We used vague uninformative priors for all parameters: uniform distributions U (-10,10) for all coefficients. Convergence was assessed by visual inspection of the chains and using the R-hat statistic (assuming no evidence of lack of convergence for values below 1.01). Models were fitted for the combined sum of the identified calls for all species combined and for each species separately. Since detectability differs among species, comparisons were made relative to each species’ activity along the transects, and not the absolute number of calls. Species richness did not differ among or within transects.

### Defining the ‘road-effect zone’

We used the change in call activity (i.e. number of calls at each survey point) with proximity to the freeway as a measure of the road-effect zone. Some national management guidelines [e.g. in the UK, 56] use a 20% decrease in activity to signify a detrimental effect on bat populations. This is a conservative measure, which is used because effects such as the road-effect zone cannot necessarily be measured directly, for example in number of individuals killed at the road. Instead, road-effect zones can result in other, indirect, impacts, such as reduced fitness or foraging success, which are harder to measure in numbers. Thus, guidelines of relative loss are conservative measures used to acknowledge when there is a greater loss in activity than can be expected by natural fluctuations in bat activity [56]. Therefore, in this study, we defined the size of the road-effect zone to be the distance from the freeway at which the activity of bats declines by 20% or more of their maximum activity along the transect monitored. Thus we: 1. determined the maximum activity per transect; 2. calculated 80% of that activity; and 3. determined the distance at which bats are 80% as active as their maximum, and therefore the distance over which activity had declined by 20% or more. We used this method to identify the size of the road-effect zone for all species combined, as well as for each individual species.

## Results

### Summary statistics

A total of 43,355 bat calls were assigned to 10 species or species complex (hereafter referred to as ‘species’ for simplicity), namely *Austronomus australis*, *Chalinolobus gouldii*, *C*. *morio*, *Nyctophilus-Myotis* complex, *Ozimops ridei*, *O*. *planiceps*, *Scotorepens balstoni*, *Vespadelus darlingtoni*, *V*. *regulus* and *V*. *vulturnus*. The mean number of identified calls per transect per night was 1204, and ranged from 47–2870 calls per transect per night. Results for each species are in S1 Table.

### Evidence of road effect zone

We found that overall bat activity decreased with increasing proximity to the freeway, showing that the freeway had a negative effect on bat activity. The estimated regression coefficients for all species combined (i.e. total activity of all species) were (numbers in parentheses indicate the limits of the 95% credible interval): ${\hat{\beta }}_{1}$ = 0.274 (0.260, 0.289) for distance from the freeway; ${\hat{\beta }}_{2}$ = -0.176 (-0.198, -0.153) for daily minimum temperature; ${\hat{\beta }}_{3}$ = -0.001 (-0.003, 0.001) for the number of large trees within a 10 m radius of the detector; and ${\hat{\beta }}_{4}$ = 0.048 (0.037, 0.058) for canopy cover. Therefore, the activity of all species combined increased with distance from the freeway and canopy cover, decreased as the daily minimum temperature decreased and did not vary with changes in number of large trees, as there were large trees along all transects (i.e. the 95% credible interval for ${\hat{\beta }}_{3}$ overlapped 0).

**Fig 2:**
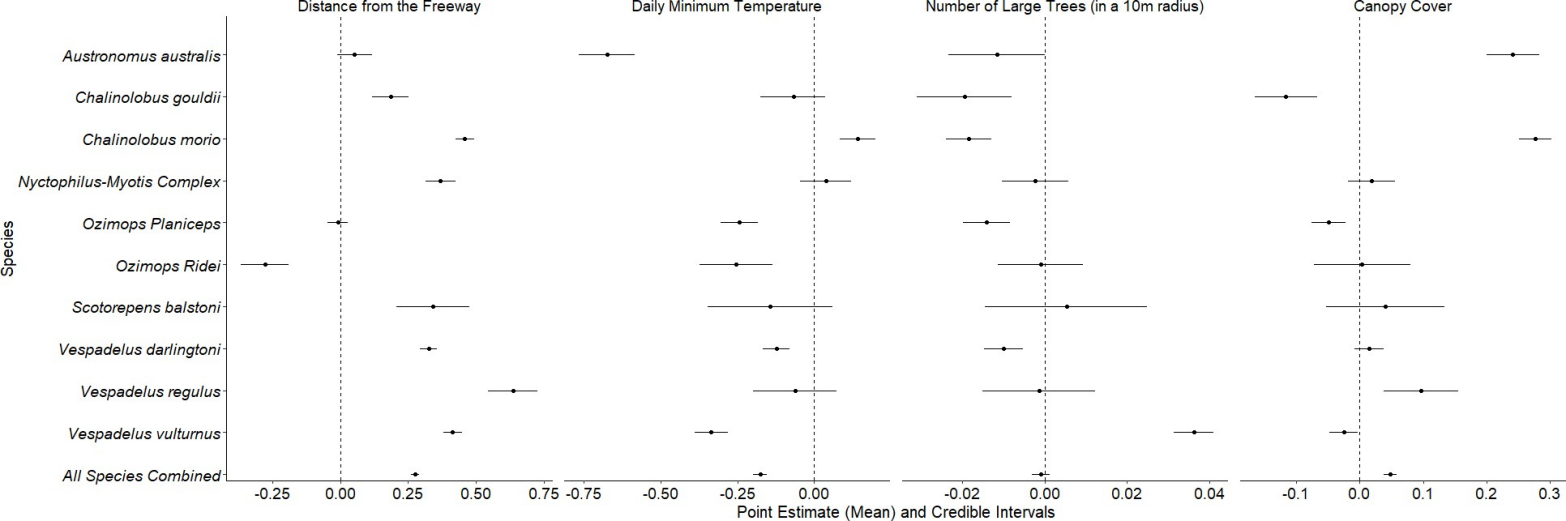
Model output. Mean point estimates and 95% credible intervals for the regression coefficients included in the model for each species of bat separately, and in the model for all species combined (“Total Call Activity”) as the response. Data collected from central Victoria, Australia. Credible intervals overlapping zero indicate coefficients that had neither positive nor negative effects on the activity of bats (at *α* = 0.05).

**Fig 3:**
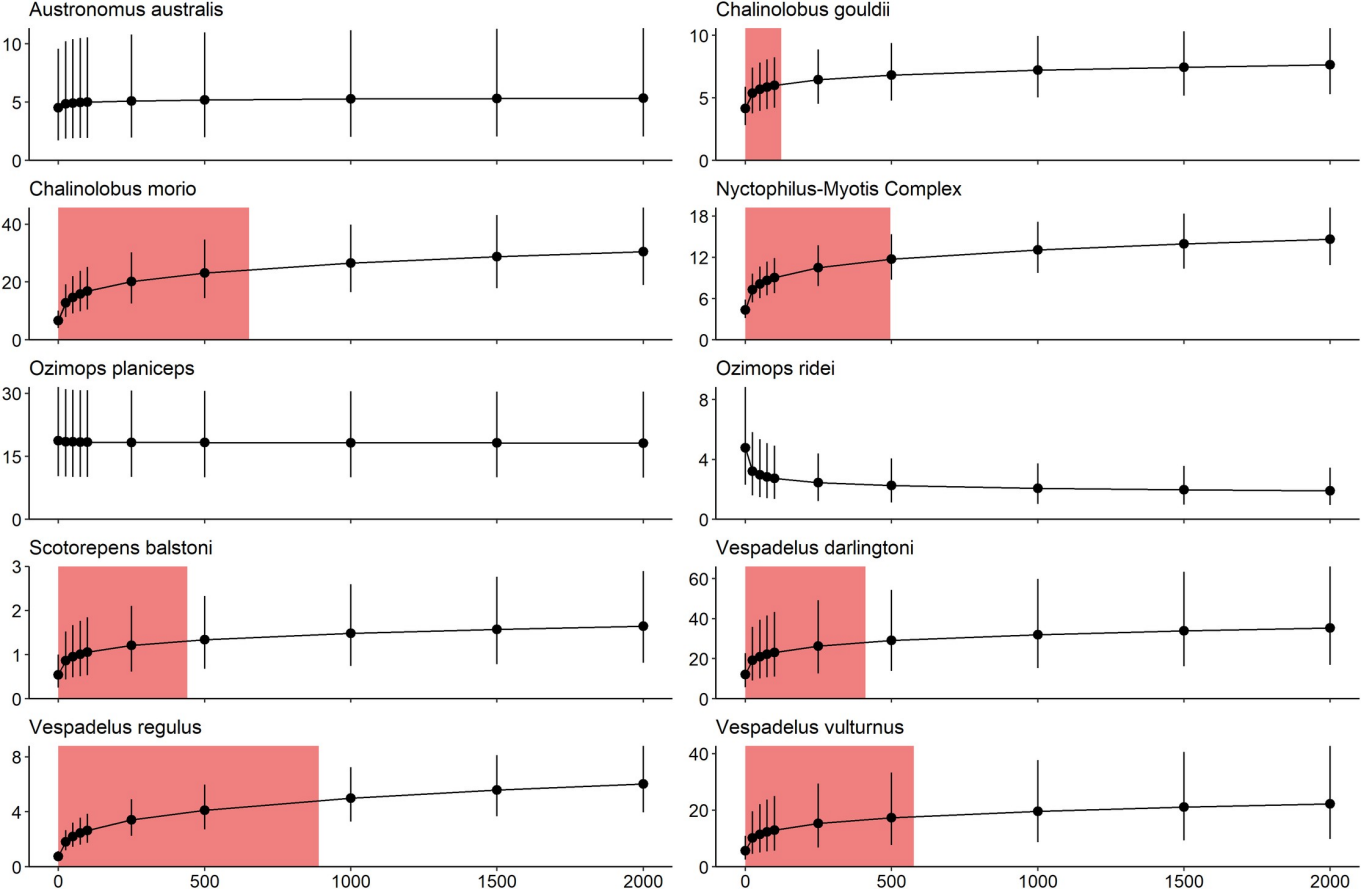
Estimated call rate for each species. Estimated mean call rate per night for each species of bat, with increasing distance from the freeway (when minimum daily temperature, number of large trees and canopy cover are held constant at their mean value). Error bars show the 95% credible interval of the estimated call rate per night. Shaded regions display the road-effect zone for each species (the distance from the freeway where activity of each species declines by at least 20%). Note the scale of the y-axes vary among plots, to assist in visualization. Data collected in central Victoria, Australia.

We identified a road effect zone (i.e. the proximity to the freeway at which call activity declines by 20% or more) for *C*. *gouldii*, *C*. *morio*, *Nyctophilus-Myotis* complex, *S*. *balstoni*, *V*. *darlingtoni*, *V*. *regulus* and *V*. *vulturnus* (Figs 2 and 3). The size of the road effect zone varied from 123 m (*C*. *gouldii*) to 890 m (*V*. *regulus*). There was no evidence of a road effect zone for *A*. *australis* and *O*. *planiceps* and conversely, *O*. *ridei*, showed a positive response to the road, with greater mean call activity closer to the freeway than further away (Figs 2 and 3).

## Discussion

### The negative road-effect zone for bats

A road-effect zone is evident when the habitat adjacent to a road supports less wildlife activity than the habitat further away. In this study, we demonstrate that bats are less active within several hundred meters of large freeways, compared to further away from freeways. Overall, the road-effect zone for the ten species we studied was 307 m wide, while the largest road-effect zone was 890 m wide for *V*. *regulus*, and the smallest road-effect zone found was 123 m wide for *C*. *gouldii*. Previous studies have demonstrated that bats in this highly cleared landscape can persist using small patches of remnant vegetation [35]. In this region, freeway verges could match this description as many have the physical characteristics of good habitat for bats–large, mature trees providing both roosting and foraging resources. However, even the smallest road-effect zone is larger than the extent of the freeway-verge (on average 28 m wide in this study). Therefore, the freeway verges in this area may not support bats as much as would be estimated by the amount of physical habitat available, and the road-effect zone may effectively reduce the amount of suitable habitat for bats in this landscape.

Traffic is likely a large contributing factor to the road-effect zone for bats [57–59]. Bats can use roadside verges in cleared landscapes when the verges are adjacent to low-traffic roads (such as the transects in this study) and railway lines [30, 60, 61], however increased traffic volume reduces bat activity in the surrounding vegetation [30, 59]. Bats are at increased risk of collisions with vehicles where linear vegetation bisects freeways [24, 26]. Furthermore, traffic noise can restrict the ability of bats to hear prey and thus reduce their foraging efficiency [28, 29, 62]. However, as the area affected by traffic noise is likely to occur in close proximity to the freeway, it is unclear what could be causing the extended road-effect zone (i.e. up to 890 m) in our study. Other factors, such as lighting from roads/vehicles, and changes in vegetation structure are less likely to influence bats in the present study because the freeways were not lit and the vegetation structure did not change along the transects. It is also unlikely that prey availability influenced the change in bat activity, since the biomass of nocturnal, flying insects does not change with distance from the freeway in this study area [63]. Therefore, the cause of the road-effect zone for bats is unclear. Future studies are needed to better understand why road-effect zones can extend hundreds of meters from a freeway, perhaps with a focus on the role road width and traffic volume play in the extent of the road-effect zone for bats.

The extent to which the freeway impacts different species of bat is highly dependent on their ecology. For example, the species that appear unaffected by the freeway, *O ridei*, *O*. *planiceps* and *A*. *australis*, tend to fly fast and high and can forage in open areas [35, 64]. As such, they may not be influenced by disturbances from the road, such as traffic noise [62, 65], like the other species which are slower, more manoeuvrable species that typically forage around trees [35]. It may also be possible that the freeway-verge in our study area can provide resources such as availability of insects [63], in habitat where there is lower competition from other bats, creating an attractive foraging resource. This highlights the importance of considering the needs of all species when undertaking protection and mitigation works, as there are inter-specific differences.

### Accounting for the road-effect zone in planning

Roads can have substantial and far-reaching impacts on the environment [4, 7–9, 20]. Quantifying the road-effect zone in management and road-planning processes is necessary to plan effectively for wildlife and their habitat requirements [66–69]. This information can help guide road-planning and decision-making on where to build roads, how to mitigate and reduce the impacts to wildlife and how much habitat is lost or degraded and needs to be compensated for. We suggest all future environmental impact assessments include quantifying the road-effect zone for sensitive wildlife, in order to best plan and mitigate the impact of roads on the environment. Furthermore, approaches to reduce the impact of the road-effect zone should be implemented. For example, creating verges with dense native vegetation may improve the quality of habitat adjacent to roads by reducing the amount of noise and light spill from traffic and lighting. The road-effect zone could also be compensated for by providing habitat further away from the freeway, which may include planting natural vegetation, or maintaining natural and unfragmented extents of available habitats. Finally, where possible, new roads should not be built in close proximity to habitat of sensitive wildlife. Incorporating road-effect zones into the environmental impact assessment for road projects will improve the chances of providing a landscape in which wildlife can persist despite human alteration of the landscape.

Identifying the extent of the road-effect zone can be difficult. For example, where species are rarely or never found near roads and only found far from roads, the presence of a road-effect zone is relatively clear. However, in situations like those presented in this study, where activity is reduced but not absent within the road-effect zone, it can be difficult to determine the biological significance of such reductions. In these situations, it is also important to consider species ecology, regular movement distances and habitual behaviours (such as a commonly used corridor etc.), as well as population viability, when identifying and mitigating the road-effect zone. Population studies could help determine if reduced activity is associated with a decline in abundance or vital rates that may affect a population’s capacity to persist within roadside environments or effectively incorporate roadside environments into their larger home ranges. As the thresholds likely vary based on type of road and the volume of traffic along those roads, a better understanding of the effect of road characteristics on the extent of the road-effect zone will help to design management strategies [2, 4, 8, 70].

Identifying a biologically relevant threshold can largely affect the recommendations and management strategies created for a given system. In this study, we used a 20% decline in activity to determine the size of the road-effect zone, based on best-practice guidelines in the UK [56]. However, had we used different thresholds, we would have arrived at very different conclusions (Table 1). For example, if the threshold was to allow a 50% decline in activity, four species *A*. *australis*, *C*. *gouldii*, *O*. *planiceps* and *O*. *ridei* would have no road-effect zone, while, the most sensitive species, *V*. *regulus* would have a road-effect zone of 161 m, compared to the 890 m we identified using the 20% decline threshold (Table 1). Alternatively, if the threshold was to allow only a 10% decline in activity, only *O*. *planiceps* and *O*. *ridei* would have no road-effect zones, while the road-effect zone for *V*. *regulus* would be up to 1365 m from the freeway (Table 1). Additionally, the threshold value can influence management decisions, for example the calculation of habitat to offset. In current practises, offsets are usually created based on the magnitude of physical habitat lost. However, offsets should also reflect habitat that may no longer support species due to the road-effect zone, despite that habitat remaining physically available. The threshold used will determine the amount of habitat affected, as well as the extent of the effect. Therefore, it is important to choose a biologically relevant threshold as it can strongly influence the estimate of the road-effect zone, and the potential consequences to management.

**Table 1:** Estimated road-effect zones for different activity thresholds.

	Extent of the road-effect zone i.e. the distance at which activity declines by the given percentage (m)					
Species or Species Complex	50%	30%	25%	20%	15%	10%
White-striped free-tailed bat–*Austronomus australis*	-	-	-	-	-	18
Gould’s wattled bat–*Chalinolobus gouldii*	-	23	55	123	263	538
Chocolate wattled bat–*Chalinolobus morio*	61	333	471	652	884	1178
Lesser long-eared bat, Gould’s long-eared bat and Large-footed myotis–*Nyctophilus-Myotis* complex	25	215	331	496	724	1035
Eastern free-tailed bat–*Ozimops ridei*	-	-	-	-	-	-
Southern free-tailed bat–*Ozimops planiceps*	-	-	-	-	-	-
Inland broad-nosed bat–*Scotorepens balstoni*	17	178	285	441	665	980
Large forest bat–*Vespadelus darlingtoni*	14	159	260	411	632	948
Southern forest bat–*Vespadelus regulus*	161	548	704	890	1109	1365
Little forest bat–*Vespadelus vulturnus*	41	274	403	577	809	1112
All species combined	5	99	178	307	511	826

The modelled road-effect zone (m) for each species, if different thresholds of activity decline (50%, 30%, 25%, 20%, 15% and 10%) are used. Distances are estimated using the model outputs. No road-effect zone reflecting the given estimated decline in activity is indicated by ‘-’. This table illustrates how the conclusions drawn regarding the extent of the road-effect zone are dependent on the thresholds set and thus these threshold values must be assigned appropriately in each road-planning project. Percentages displayed are chosen to be illustrative of the differences.

## Conclusion

Habitat indirectly lost or degraded within the road-effect zone can substantially change estimates of the amount of habitat available for wildlife to occupy. In this study, we quantified a negative road-effect zone, at least 123 m wide, for seven out of 10 species of insectivorous bats in southeast Australia. The road-effect zone for bats was overwhelmingly negative, and should therefore be considered in road-planning projects. Evaluating the road-effect zone early in environmental assessment processes can help guide decisions on road planning, building and mitigation strategies. Understanding the mechanisms and biological consequences of the road-effect zone can help develop appropriate and targeted strategies to reduce the impacts. Roads fragment much of our earth and understanding their full ecological impact is essential to maintain thriving wildlife populations and functioning ecosystems.

## Supporting information

S1 Table Summary of data collected. Mean, median and range of number of bat calls per transect (combining 10 sampling points along each transect), per night for each species or species complex, across all three freeways (Hume Freeway, Calder Freeway and Goulburn Valley Freeway). Calls were collected at eighteen transects, over two consecutive nights at each transect.
(DOCX)

## References

[1] Riitters KH, Wickham JD. How far to the nearst road? Frontiers in Ecology and the Environment. 2003;1:125–9.

[2] Fahrig L, Rytwinski T. Effects of roads on animal abundance: An empirical review and synthesis. Ecology and Society. 2009;14(1):21. PubMed PMID: WOS:000267846300048.

[3] Forman RTT, Alexander LE. Roads and their major ecological effects. Annual Review of Ecology and Systematics. 1998;29(1):207–31. 10.1146/annurev.ecolsys.29.1.207

[4] Spellerberg IF. Ecological effects of roads and traffic: a literature review. Global Ecology and Biogeography. 1998;7(5):317–33. 10.2307/2997681 PubMed PMID: WOS:000077864500001.

[5] van der Ree R, Smith DJ, Grilo C. Handbook of Road Ecology. Chichester, West Sussex: John Wiley & Sons, Ltd; 2015.

[6] Bennett VJ. Effects of road density and pattern on the conservation of species and biodiversity. Current Landscape Ecology Reports. 2017;2(1):1–11. 10.1007/s40823-017-0020-6

[7] Forman RTT, Sperling D, Bissonette JA, Clevenger AP, Cutshall CD, Dale VH, et al. Road Ecology: Science and Solutions. Washington, DC, USA: Island Press; 2003.

[8] Trombulak SC, Frissell CA. Review of ecological effects of roads on terrestrial and aquatic communities. Conservation Biology. 2000;14(1):18–30. 10.1046/j.1523-1739.2000.99084.x PubMed PMID: WOS:000085366700006.

[9] Coffin AW. From roadkill to road ecology: A review of the ecological effects of roads. Journal of Transport Geography. 2007;15(5):396–406. 10.1016/j.jtrangeo.2006.11.006 PubMed PMID: WOS:000249413200007.

[10] Forman RTT, Reineking B, Hersperger AM. Road traffic and nearby grassland bird patterns in a suburbanizing landscape. Journal of Environmental Management. 2002;29(6):782–800. Epub 2002/05/07. 10.1007/s00267-001-0065-4 . 11992171

[11] Kuitunen MT, Viljanen J, Rossi E, Stenroos A. Impact of busy roads on breeding success in pied flycatchers *Ficedula hypoleuca*. Journal of Environmental Management. 2003;31(1):79–85. Epub 2002/11/26. 10.1007/s00267-002-2694-7 . 12447577

[12] Shanley CS, Pyare S. Evaluating the road-effect zone on wildlife distribution in a rural landscape. Ecosphere. 2011;2(2):1–16. 10.1890/ES10-00093.1

[13] Zurcher AA, Sparks DW, Bennett VJ. Why the bat did not cross the road? Acta Chiropterologica. 2010;12(2):337–40. 10.3161/150811010x537918 PubMed PMID: WOS:000285772400008.

[14] Berthinussen A, Altringham J. The effect of a major road on bat activity and diversity. Journal of Applied Ecology. 2012;49(1):82–9. 10.1111/j.1365-2664.2011.02068.x PubMed PMID: WOS:000299153800011.

[15] Pellet J, Guisan A, Perrin N. A concentric analysis of the impact of urbanization on the threatened European tree frog in an agricultural landscape. Conservation Biology. 2004;18(6):1599–606. 10.1111/j.1523-1739.2004.0421a.x PubMed PMID: WOS:000225737300023.

[16] Semlitsch RD, Ryan TJ, Hamed K, Chatfield M, Drehman B, Pekarek N, et al. Salamander abundance along road edges and within abandoned logging roads in Appalachian forests. Conservation Biology. 2007;21(1):159–67. Epub 2007/02/15. 10.1111/j.1523-1739.2006.00571.x . 17298522

[17] Eigenbrod F, Hecnar SJ, Fahrig L. Quantifying the road-effect zone: Threshold effects of a motorway on anuran populations in Ontario, Canada. Ecology and Society. 2009;14(1):24. 10.5751/es-02691-140124 PubMed PMID: WOS:000267846300015.

[18] Boarman WI, Sazaki M. A highway’s road-effect zone for desert tortoises (Gopherus agassizii). Journal of Arid Environments. 2006;65(1):94–101. 10.1016/j.jaridenv.2005.06.020 PubMed PMID: WOS:000235767200006.

[19] Tanner D, Perry J. Road effects on abundance and fitness of Galapagos lava lizards (Microlophus albemarlensis). Journal of Environmental Management. 2007;85(2):270–8. Epub 2007/01/09. 10.1016/j.jenvman.2006.08.022 . 17208350

[20] Benitez-Lopez A, Alkemade R, Verweij PA. The impacts of roads and other infrastructure on mammal and bird populations: A meta-analysis. Biological Conservation. 2010;143(6):1307–16. 10.1016/j.biocon.2010.02.009 PubMed PMID: WOS:000278572300001.

[21] Forman RTT. Estimate of the area affected ecologically by the road system in the United States. Conservation Biology. 2000;14(1):31–5. 10.1046/j.1523-1739.2000.99299.x PubMed PMID: WOS:000085366700007.

[22] Sih A, Jonsson BG, Luikart G. Habitat loss: ecological, evolutionary and genetic consequences. Trends in Ecology & Evolution. 2000;15(4):132–4. 10.1016/s0169-5347(99)01799-1

[23] Kitzes J, Merenlender A. Large roads reduce bat activity across multiple species. PLoS One. 2014;9(5):e96341. Epub 2014/05/16. 10.1371/journal.pone.0096341 24823689 PMC4019470

[24] Fensome AG, Mathews F. Roads and bats: a meta-analysis and review of the evidence on vehicle collisions and barrier effects. Mammal Review. 2016;46(4):311–23. Epub 2016/10/07. 10.1111/mam.12072 27708480 PMC5031215

[25] Hale JD, Fairbrass AJ, Matthews TJ, Davies G, Sadler JP. The ecological impact of city lighting scenarios: exploring gap crossing thresholds for urban bats. Global Change Biology. 2015;21:2467–78. Epub 2015/02/04. 10.1111/gcb.12884 25644403 PMC4975606

[26] Bennett VJ, Zurcher AA. When corridors collide: road-related disturbance in commuting bats. Journal of Wildlife Management. 2013;77(1):93–101. 10.1002/jwmg.467 PubMed PMID: WOS:000313118400013.

[27] Stone EL, Harris S, Jones G. Impacts of artificial lighting on bats: a review of challenges and solutions. Mammalian Biology. 2015;80(3):213–9. 10.1016/j.mambio.2015.02.004 PubMed PMID: WOS:000356738900009.

[28] Schaub A, Ostwald J, Siemers BM. Foraging bats avoid noise. Journal of Experimental Biology. 2008;211(Pt 19):3174–80. Epub 2008/09/23. 10.1242/jeb.022863 . 18805817

[29] Siemers BM, Schaub A. Hunting at the highway: traffic noise reduces foraging efficiency in acoustic predators. Proceedings of the Royal Society B: Biological Sciences. 2011;278(1712):1646–52. Epub 2010/11/19. 10.1098/rspb.2010.2262 21084347 PMC3081776

[30] Medinas D, Ribeiro V, Marques JT, Silva B, Barbosa AM, Rebelo H, et al. Road effects on bat activity depend on surrounding habitat type. Science of the Total Environment. 2019;660:340–7. Epub 2019/01/15. 10.1016/j.scitotenv.2019.01.032 . 30640102

[31] Claireau F, Bas Y, Pauwels J, Barré K, Machon N, Allegrini B, et al. Major roads have important negative effects on insectivorous bat activity. Biological Conservation. 2019;235:53–62. 10.1016/j.biocon.2019.04.002

[32] Russo D, Ancillotto L. Sensitivity of bats to urbanization: a review. Mammalian Biology. 2015;80(3):205–12. 10.1016/j.mambio.2014.10.003 PubMed PMID: WOS:000356738900008. 32226358 PMC7094881

[33] Park KJ. Mitigating the impacts of agriculture on biodiversity: bats and their potential role as bioindicators. Mammalian Biology. 2015;80(3):191–204. 10.1016/j.mambio.2014.10.004 PubMed PMID: WOS:000356738900007.

[34] Jones G, Jacobs DS, Kunz TH, Willig MR, Racey PA. Carpe noctem: the importance of bats as bioindicators. Endangered Species Research. 2009;8:93–115. 10.3354/esr00182

[35] Lumsden LF, Bennett AF. Scattered trees in rural landscapes: foraging habitat for insectivorous bats in south-eastern Australia. Biological Conservation. 2005;122(2):205–22. 10.1016/j.biocon.2004.07.006 PubMed PMID: WOS:000225818100005.

[36] Costermans L. Trees of Victoria and Adjoining Areas. Frankston, Victoria, Australia: Costermans Publishing; 2006.

[37] Agriculture Victoria. Primary Production Landscapes of Victoria—Central Victoria: State of Victoria; 2020. Available from: http://vro.agriculture.vic.gov.au /dpi/vro/vrosite.nsf/pages/primary_prod_landscapes_central_vic.

[38] VicRoads. VicRoads Open Data Site. 2015 ed. http://vicroadsopendata.vicroadsmaps. opendata.arcgis.com/2015.

[39] Lumsden L, Bennett A, Krasna S, Silins J. The conservation of insectivorous bats in rural landscapes of northern Victoria. In: Bennett AF, Backhouse G, Clark T, editors. People and Nature Conservation: Perspectives on Private Land Use and Endangered Species Recovery: Royal Zoological Society of New South Wales: Sydney; 1995.

[40] Lumsden LF, Bennett AF, Silins JE. Location of roosts of the lesser long-eared bat *Nyctophilus geoffroyi* and Gould’s wattled bat *Chalinolobus gouldii* in a fragmented landscape in south-eastern Australia. Biological Conservation. 2002;106(2):237–49. doi: Pii S0006-3207(01)00250-6 10.1016/S0006-3207(01)00250-6 PubMed PMID: WOS:000176269900010.

[41] Lumsden LF, Bennett AF, Silins JE. Selection of roost sites by the lesser long-eared bat (Nyctophilus geoffroyi) and Gould’s wattled bat (Chalinolobus gouldii) in south-eastern Australia. Journal of Zoology. 2002;257(2):207–18. Epub 2002/06/17. 10.1017/S095283690200081X

[42] Lentini PE, Gibbons P, Fischer J, Law B, Hanspach J, Martin TG. Bats in a farming landscape benefit from linear remnants and unimproved pastures. PLoS One. 2012;7(11):e48201. Epub 2012/11/17. 10.1371/journal.pone.0048201 23155378 PMC3498260

[43] Downs NC, Racey PA. The use by bats of habitat features in mixed farmland in Scotland. Acta Chiropterologica. 2006;8(1):169–85. 10.3161/1733-5329(2006)8[169:Tubboh]2.0.Co;2

[44] Murray SW, Kurta A. Nocturnal activity of the endangered Indiana bat (Myotis sodalis). Journal of Zoology. 2004;262(2):197–206. Epub 2004/01/19. 10.1017/S0952836903004503

[45] Russo D, Jones G, Migliozzi A. Habitat selection by the Mediterranean horseshoe bat, Rhinolophus euryale (Chiroptera: Rhinolophidae) in a rural area of southern Italy and implications for conservation. Biological Conservation. 2002;107(1):71–81.

[46] Gibson M, Lumsden L. The Anascheme automated bat call identification system. The Australasian Bat Society Newsletter. 2003;20:24–6.

[47] Adams MD, Law BS, Gibson MS. Reliable automation of bat call identification for eastern New South Wales, Australia, using classification trees and AnaScheme software. Acta Chiropterologica. 2010;12(1):231–45. 10.3161/150811010x504725 PubMed PMID: WOS:000278753700018.

[48] Medinas D, Marques JT, Mira A. Assessing road effects on bats: the role of landscape, road features, and bat activity on road-kills. Ecological Research. 2013;28(2):227–37. 10.1007/s11284-012-1009-6 PubMed PMID: WOS:000316002800012.

[49] O’Donnell CFJ. Influence of season, habitat, temperature, and invertebrate availability on nocturnal activity of the New Zealand long‐tailed bat (Chalinolobus tuberculatus). New Zealand Journal of Zoology. 2000;27(3):207–21. 10.1080/03014223.2000.9518228

[50] Anthony E, Stack M, Kunz T. Night roosting and the nocturnal time budget of the little brown bat, Myotis lucifugus: effects of reproductive status, prey density, and environmental conditions. Oecologia. 1981;51(2):151–6. 10.1007/BF00540593 28310074

[51] Richards G. Nocturnal activity of insectivorous bats relative to temperature and prey availability in tropical Queensland. Wildlife Research. 1989;16(2):151–8.

[52] Rydell J. Feeding activity of the northern bat *Eptesicus nilssoni* during pregnancy and lactation. Oecologia. 1989;80(4):562–5. Epub 1989/09/01. 10.1007/BF00380082 . 28312844

[53] Plummer M. JAGS: A program for analysis of Bayesian graphical models using Gibbs sampling. Proceedings of the 3rd International Workshop on Distributed Statistical Computing. 2003.

[54] R Core Team. R: A language and environment for statistical computing. 3.3.2 ed. Vienna, Austria: R Foundation for Statistical Computing; 2019.

[55] Su Y-S, Yajima M. R2jags: Using R to Run ’JAGS’. R package version 0.5–7. 2015.

[56] Berthinussen A, Altringham J. WC1060 Development of a cost-effective method for monitoring the effectiveness of mitigation for bats crossing linear transport infrastructure. UK: Depratment for Environmental Food and Rural Affairs, 2015.

[57] Abbott IM, Butler F, Harrison S. When flyways meet highways–The relative permeability of different motorway crossing sites to functionally diverse bat species. Landscape and Urban Planning. 2012;106(4):293–302. 10.1016/j.landurbplan.2012.03.015

[58] Bennett VJ, Sparks DW, Zollner PA. Modeling the indirect effects of road networks on the foraging activities of bats. Landscape Ecology. 2013;28(5):979–91. 10.1007/s10980-013-9874-0 PubMed PMID: WOS:000318494500014.

[59] Borkin KM, Smith DH, Shaw WB, McQueen JC. More traffic, less bat activity: the relationship between overnight traffic volumes and Chalinolobus tuberculatus activity along New Zealand highways. Acta Chiropterol. 2019;21(2):321–9.

[60] Lumsden LF, Bennett AF. Bats in rural landscapes: a significant but largely unknown faunal component. In: Barlow T, Thornburn R, editors. the Bushcare Grassy Landscapes Conference. Clare, South Australia: Environment Australia, Canberra.; 2000.

[61] Vandevelde J-C, Bouhours A, Julien J-F, Couvet D, Kerbiriou C. Activity of European common bats along railway verges. Ecological Engineering. 2014;64:49–56. 10.1016/j.ecoleng.2013.12.025

[62] Bonsen G, Law B, Ramp D. Foraging strategies determine the effect of traffic noise on bats. Acta Chiropterologica. 2015;17(2):347–57. 10.3161/15081109acc2015.17.2.010 PubMed PMID: WOS:000370996300010.

[63] Bhardwaj M, Soanes K, Lahoz-Monfort JJ, Lumsden LF, van der Ree R. Little evidence of a road-effect zone for nocturnal, flying insects. Ecology and Evolution. 2019;9(1):65–72. Epub 2019/01/27. 10.1002/ece3.4609 30680096 PMC6342180

[64] Reside AE, Lumsden LF. Resource partitioning by two closely-related sympatric freetail bats, Mormopterus spp. In: Law B, Eby P, Lunney D, Lumsden L, editors. Biology and Conservation of Australasian Bats: Royal Zoological Society New South Wales: Sydney; 2011. p. 155–66.

[65] Bhardwaj M, Soanes K, Straka TM, Lahoz-Monfort JJ, Lumsden LF, van der Ree R. Differential use of highway underpasses by bats. Biological Conservation. 2017;212:22–8. 10.1016/j.biocon.2017.05.022 PubMed PMID: WOS:000407186000003.

[66] Iuell B, Bekker H, Cuperus R, Dufek J, Fry GL, Hicks C, et al., editors. Wildlife and Traffic—A European Handbook for Identifying Conflicts and Designing Solutions. Prepared by COST 341—Habitat Fragmentation due to Transportation Infrastructure. Delft, The Netherlands: Ministry of Transport, Public Works and Water Management, Road and Hydraulic Engineering division, Delft, The Netherlands.; 2003.

[67] Roberts K, Sjölund A. Incorporating biodiversity issues into road design: The road agency perspective. In: van der Ree R, Smith DJ, Grilo C, editors. Handbook of Road Ecology. Chichester, West Sussex: John Wiley & Sons, Ltd.; 2015. p. 27–31.

[68] Rytwinski T, Fahrig L. The Impacts of Roads and Traffic on Terrestrial Animal Populations. In: van der Ree R, Smith DJ, Grilo C, editors. Handbook of Road Ecology. Chichester, West Sussex: John Wiley & Sons, Ltd; 2015. p. 237–46. 10.1016/j.jenvman.2015.01.048

[69] van der Ree R, Smith DJ, Grilo C. The ecological effects of linear infrastructure and traffic: challenges and opportunites of rapid global growth. In: van der Ree R, Smith DJ, Grilo C, editors. Handbook of Road Ecology. Chichester, West Sussex: John Wiley & Sons; 2015. p. 1–9.

[70] Forman RT, Deblinger RD. The ecological road‐effect zone of a Massachusetts (USA) suburban highway. Conservation biology. 2000;14(1):36–46.

