# Supplementary material for: Insectivorous bats are less active near freeways
Source: PLoS One. 2021 Mar 10;16(3):e0247400. doi: 10.1371/journal.pone.0247400 (PMC7946297; doi:10.1371/journal.pone.0247400)
Supplement: S1 Table — Mean, median and range of number of bat calls per transect (combining 10 sampling points along each transect), per night for each species or species complex, across all three freeways (Hume Freeway, Calder Freeway and Goulburn Valley Freeway). Calls were collected at eighteen transects, over two consecutive nights at each transect. (DOCX) [file pone.0247400.s001.docx]

S1 Table. Summary of data collected. Mean, median and range of number of bat calls per transect (combining 10 sampling points along each transect), per night for each species or species complex, across all three freeways (Hume Freeway, Calder Freeway and Goulburn Valley Freeway). Calls were collected at eighteen transects, over two consecutive nights at each transect.

| Species or Species Complex | Mean | Median | Range |
| --- | --- | --- | --- |
| White-striped free-tailed bat – *Austronomus australis* | 68 | 30 | 0 – 389 |
| Gould’s wattled bat – *Chalinolobus gouldii* | 53 | 50 | 3 – 220 |
| Chocolate wattled bat *– Chalinolobus morio* | 213 | 157 | 5 – 892 |
| Lesser long-eared bat, Gould’s long-eared bat and Large-footed myotis – *Nyctophilus-Myotis* complex | 91 | 76 | 7 – 290 |
| Eastern free-tailed bat – *Ozimops ridei* | 37 | 19 | 0 – 174 |
| Southern free-tailed bat – *Ozimops planiceps* | 183 | 115 | 6 – 730 |
| Inland broad-nosed bat – *Scotorepens balstoni* | 15 | 9 | 0 – 52 |
| Large forest bat – *Vespadelus darlingtoni* | 274 | 224 | 0 – 1054 |
| Southern forest bat – *Vespadelus regulus* | 35 | 27 | 1 – 106 |
| Little forest bat – *Vespadelus vulturnus* | 227 | 156 | 0 – 817 |
| All species combined | 1204 | 1081 | 47 – 2870 |
